# Mechanical Properties and Corrosion Resistance of NbTiAlSiZrN_x_ High-Entropy Films Prepared by RF Magnetron Sputtering

**DOI:** 10.3390/e21040396

**Published:** 2019-04-13

**Authors:** Qiuwei Xing, Haijiang Wang, Mingbiao Chen, Zhaoyun Chen, Rongbin Li, Peipeng Jin, Yong Zhang

**Affiliations:** 1 State Key Laboratory of Advanced Metals and Materials, University of Science and Technology Beijing, Beijing 100083, China; 2 School of Mechanical Engineering, Qinghai University, Xining 810016, China; 3 Qinghai Provincial Engineering Research Center of High Performance Light Metal Alloys and Forming, Xining 810016, China; 4 Qinghai Provincial Key Laboratory of New Light Alloys, Xining 810016, China; 5 University of Sheffield, Sheffield S37GE, UK; 6 School of Materials Science, Shanghai DianJi University, Shanghai 201306, China

**Keywords:** thin films, high-entropy alloy films, hardness, corrosion-resistance

## Abstract

In this study, we designed and fabricated NbTiAlSiZrN_x_ high-entropy alloy (HEA) films. The parameters of the radio frequency (RF) pulse magnetron sputtering process were fixed to maintain the N_2_ flux ratio at 0%, 10%, 20%, 30%, 40%, and 50%. Subsequently, NbTiAlSiZrN_x_ HEA films were deposited on the 304 stainless steel (SS) substrate. With an increasing N_2_ flow rate, the film deposited at a R_N_ of 50% had the highest hardness (12.4 GPa), the highest modulus (169 GPa), a small roughness, and a beautiful color. The thicknesses of the films were gradually reduced from 298.8 nm to 200 nm, and all the thin films were of amorphous structure. The electrochemical corrosion resistance of the film in a 0.5 mol/L H_2_SO_4_ solution at room temperature was studied and the characteristics changed. The HEA films prepared at N_2_ flow rates of 10% and 30% were more prone to corrosion than 304 SS, but the corrosion rate was lower than that of 304 SS. NbTiAlSiZrN_x_ HEA films prepared at N_2_ flow rates of 20%, 40%, and 50% were more corrosion-resistant than 304 SS. In addition, the passivation stability of the NbTiAlSiZrN_x_ HEA was worse than that of 304 SS. Altogether, these results show that pitting corrosion occurred on NbTiAlSiZrN_x_ HEA films.

## 1. Introduction

Improving corrosive resistance and mechanical properties is an important part of alloy thin film research [1,2,3,4,5,6,7]. The reasonable design of alloy thin films is the premise for the preparation of excellent films [8,9,10], whereas the proper preparation of the target and selection of the appropriate substrate are prerequisites in their preparation [11,12]. High-entropy alloys (HEAs) have excellent performance due to their high mixing entropy effects, microstructure strengthening, sluggish diffusion, cocktail effects, and structural stability effects [13,14,15,16]. Feng et al. reported that the mechanical property of FeCoNi(AlSi)_x_ is affected by the chemical short-range order in alloys [17]. Adding Co and Gd can affect the microstructures and properties of FeSiBAlNi HEAs [18]. The oxide can improve the tensile strength of CrMnFeCoNi HEAs [19]. Besides composition, fabrication methods can also affect the microstructure and mechanical properties of HEAs [20,21]. Magnetron sputtering and laser cladding are two of the most important methods for preparing HEA coatings [22,23,24,25,26,27,28]. Both N_2_ flow rate and substrate bias affect hardness, the elastic modulus, and corrosive resistance of HEA coatings. Hsueh et al. prepared a (AlCrSiTiZr)_(100-x)_N_x_ coating (amorphous) by magnetron sputtering on the surface of a 6061 aluminum alloy plate. The researchers changed the conditions of nitrogen flow ratio (R_N_) to increase the (AlCrSiTiZr)_100-x_N_x_ thin film content to 30%, which was deposited on the 6061 aluminum alloy and carbon steel substrate, thereby optimizing the mechanical properties and corrosive resistance of HEA thin films. Hardness of the HEA (AlCrSiTiZr)_(100-x)_N_x_ coating (amorphous) reaches 34 GPa [7], while that of NiCrAlCoMo HEA thin films was 1000 HV. The films were prepared by magnetron sputtering on a carbon steel substrate, and contained more intermetallic compounds. Its friction coefficient was low at 0.6, and it had good wear resistance. The hardness of the magnetron sputtering (AlCrNbSiTiV)N HEA film was 41 GPa. The hardness of the (AlCrTiZr)Si_7.9_N film was 34 GPa, and the oxidation resistance is excellent [29]. Hardness of the HEA film (AlCrTiZr) N_0.6_C_0.2_ was 32 GPa, and the creep rate was low at 1.1 × 10^−4^ s^−1^. The corrosive resistance was excellent [30]. The Al_x_FeCrCoNiCu HEA coating prepared on a steel substrate by magnetron sputtering has good anti-corrosive properties [31]. Sheng et al. [32] used direct-current (DC) bias magnetron sputtering amorphous NbTiAlSiNx films with high hardness and modulus. The microstructures of the films are amorphous, and the microstructures are transformed into crystal structures at temperatures above 1000 °C. Sheng et al. [23] reported that amorphous NbTiAlSiW_x_N_y_ HEA films have high hardness and modulus, and are thermally stabile up to 700 °C, whereas the films transformed into crystal structures at temperatures greater than 1000 °C. Xing et al. [33] reported that NbTiAlSiZrN_x_ HEA films sputtered by direct current power had good thermal stability at 600 °C. The Cr_6_Fe_6_V_6_Ta_42_W_40_ multicomponent alloy film exhibited great solar absorption at room temperature [34]. The (Al_0.5_CrFeNiTi_0.25_)N_x_ HEA nitride films achieved higher hardness and Young’s modulus than as-cast (Al_0.5_CrFeNiTi_0.25_)N_x_ bulk alloys [35]. These results indicated that HEA films have potential application on protective coatings to improve the hardness and corrosion resistance of knives and cutting tools. 

TiAlN and ZrN films have been widely used as protective coating. The NbTiAlSi system HEA films have been reported to possess high hardness and modulus [23,32,33]. CoCrFeNiAl_0.3_ films fabricated by RF sputtering demonstrate better corrosion resistance than in 3.5 wt. % NaCl solution. The hardness of CoCrFeNiAl_0.3_ can reach 11.5 GPa. Moreover, the addition of non-metallic elements has been shown to be an effective means for improving the properties of HEAs [36]. In this regard, we prepared a new five primary element NbTiAlSiZr HEA of equimolar content, and then used the processing methods and R_N_ conditions—which were different from those previously reported in [25], using the radio frequency (RF) magnetron sputtering equipment, processes, and other parameters of fixed technologies—to prepare the NbTiAlSiZrN_x_ HEA thin films under different R_N_ values (0%, 10%, 20%, 30%, 40%, and 50%) and gases (i.e., R_N_ is the only process variable). Then the roughness, color, and performance of these thin films were studied. Spectroscopic analysis showed that the NbTiAlSiZrN_x_ HEA thin film chemical composition was feasible, the preparation process was appropriate, the phase structure was amorphous, and the performance testing indicators met the requirements. Our data show that with increasing R_N_, the film’s performance indicators, surface color, and roughness could be changed.

## 2. Materials and Methods 

### 2.1. Design and Fabrication of NbTiAlSiZrN_x_ HEA Thin Films

#### 2.1.1. Design of NbTiAlSiZrN_x_ HEA Films

According to the theory and design of HEAs, as well as the results of previous studies on NbTiAlSiZr HEA synthesis, the elemental composition of Nb:Ti:Al:Si:Zr was 20:20:20:20:20. 

According to Equations (1) and (2), we calculated the radius difference of the constituent atoms as follows:

(1) $$\delta =\sqrt{\overset{n}{\underset{i=1}{\sum }}c_{i}{\left(1-\frac{r_{i}}{\bar{r}}\right)}^{2}}$$

(2) $$\bar{r}=\sum_{i=1}^{n}c_{i}r_{i}$$

where $\bar{r}$ is the average radius of alloying elemental atoms and *r_i_* is the radius of the *i*th elemental atom (Table 1). 

The calculated results (*δ* = 1.1% < 6.5%) reflected a change in the atomic radius of the primary element after HEA formation.

According to Equation (3), we calculated the mixing enthalpy Δ*S_mix_* of the primary elements Nb, Ti, Al, Si, and Zr as follows:

(3) $$\Delta S_{mix}=-R\overset{n}{\underset{i=1}{\sum }}\left(c_{i}\ln c_{i}\right)$$

where R = 8.314 J/mol·K is the gas constant, *n* is the primary element number of the alloy, and *c_i_* is the *i*th primary element content (%).

The calculated results (Δ*S_mix_* = 1.566 R) failed to meet the thermodynamic conditions (1 R < Δ*S_mix_* < 1.5 R) of the solid HEA. These results suggest that an amorphous or compound HEA was formed. The NbTiAlSiZrN_x_ HEA film had a thickness of 200–300 nm and a surface roughness of 0.2–0.4 nm.

#### 2.1.2. Preparing the NbTiAlSiZrN_x_ HEA Film

The commonly used methods for preparing HEA films are co-sputtering with multi-target and sputtering by HEA target. The compositional deviation of the latter method is less than the former. In this regard, we sputtered the HEA target to form HEA films. According to the constituent elements of the NbTiAlSiZr HEA, the HEA target was prepared using a hot isostatic pressing process. In brief, different NbTiAlSiZrN_x_ HEA films were prepared using a JGP-450 single-target RF magnetron sputtering instrument with parameters as follows: a target base distance of 60 mm; a vacuum pressure of 6 × 10^−4^ Pa; a sputtering pressure of 0.6 Pa; an Ar gas flow rate constant of 25 sccm; a voltage of 600 V; a current of 180 mA; a sputtering power of 120 W; a pulse frequency of 13.56 MHz; and nitrogen flow rates of 0, 3, 6, 11, 17, and 25 sccm, respectively (i.e., corresponding to nitrogen flow rates of 0, 10, 20, 30, 40, and 50%, respectively), and a single sample sputtering time of 60 min. The elemental components of the HEA film were identified by energy dispersive X-Ray spectroscopy (EDX) after sputtering. For the potential compositional deviation, we prepared two solutions: (1) using assisted targets to supplement the lack elements; and (2) adjusting the composition of the alloy target. The elemental components of NbTiAlSiZr HEA films are listed in Table 2. According to Table 2, the maximum element deviation is only 1 at. % after testing (Al: 21.00 at. %). Because the accuracy range of EDX measurements is not less than 1 at. %, the composition of HEA film was uniform and did not need further compositional adjustment in this work.

### 2.2. Characterization of the NbTiAlSiZrNx HEA Thin Film Method

#### 2.2.1. Assessment of Film Thickness

The film cross-sections were observed under an Auriga model field emission scanning electron microscope (Carl Zeiss, Jena, Germany). Working distance (WD) was 9 mm and the extra high tension (EHT) was 15 kV.

Based on our experience with alloys, we opted to use N-type single-side polished monocrystalline silicon (100) with a purity of 99.999% and a size of φ 50.8 × 0.5 mm as the substrate. The size of the single crystal sodium chloride ([100] crystal plane) was 20 × 20 × 2 mm, whereas that of the single-side polished 304 stainless steel (304 SS) was 10 × 10 × 2 mm. Single crystal silicon ([100] crystal face) substrate films are conducive to tests of phase structure, whereas single crystal sodium chloride ([100] crystal face) substrate films are conducive to transmission analyses of the phase structure. In addition, 304 stainless steel (304 SS) substrate films are conducive to testes of corrosion resistance. An ultrasonic BG-01 model cleaner was used to clean the substrates, followed by drying.

#### 2.2.2. Assessment of the Film Surface

The film surface was observed under a Bruker multi-mode 8 SPM atomic force microscope, and the film surface roughness was measured in the Scan Asyst smart imaging mode. The size of scanning range was 5 × 5 μm^2^.

#### 2.2.3. Assessment of Film Hardness

The displacement-load curves of the thin film were measured with a nano-indentor, and then the hardness and modulus were calculated. The working parameters were as follows: tan effective range of 100–200 nm, a press-in depth of 1 μm, a press-in rate of 0.05 nm/s, and Poisson’s ratio of 0.25. 

#### 2.2.4. Thin Film Corrosion Resistance

Electrochemical potentiometric electrode polarization tests were performed on thin film-coated 304 SS substrates using the PARSTAT Model 2273 electrochemical workstation at room temperature. The saturated calomel electrode served as the reference electrode, and the platinum electrode served as the auxiliary electrode. The thin film-coated 304 SS substrate was the working electrode, and the etching solution was 0.5 mol/L H_2_SO_4_. The sample that was submerged into the corrosive solution was connected to a wire, and all other bare surfaces excluded from the test surface were coated with silica gel. The open-circuit potential of the sample was measured until it stabilized, followed by scanning of the potential. The range of the potential scan was −1.0–1.5 V, scanning speed was 1 mV/s, and samples were etched for 2500 s in 0.5 mol/L H_2_SO_4_ solution.

#### 2.2.5. Thin Film Crystal Structure Test

The surface morphology of the thin film was observed under a Technic G2 F-30 S-TWIN transmission electron microscope (TEM). The sample, with a size of 5 × 5 mm, was coated onto the sodium chloride substrate. The sodium chloride substrate was dissolved in deionized water to obtain a thin film for observation using TEM. The sample was measured with an O8DISCOVER high-resolution diffractometer. The sample size was 10 × 10 mm, and EHT was 300 kV.

The crystal structure of the thin film was determined using a Dmax X-ray diffractometer and a Cu Kα (40 kV, 20 mA) source. The X-ray grazing angle was 1°, the diffraction angle 2θ range was 20–80°, and the scanning speed was 3 °/s.

## 3. Results

### 3.1. Assessment of Film Thickness

**Figure 1:**
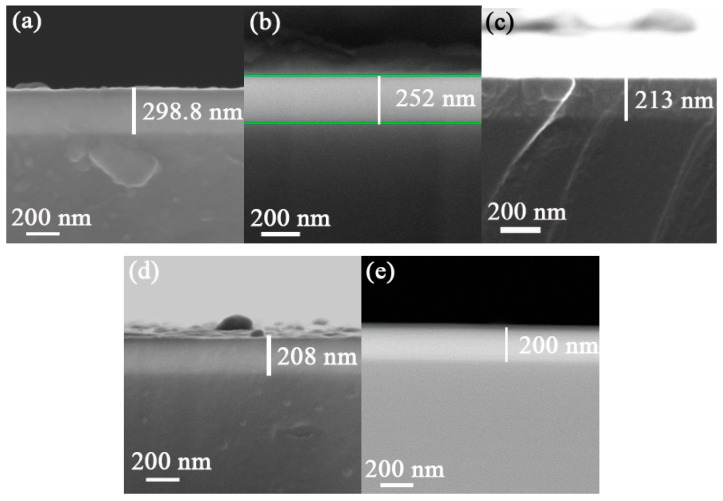
SEM images of NbTiAlSiZrN_x_ HEA films’ morphology at: (**a**) R_N_ = 10%, (**b**) R_N_ = 20%, (**c**) R_N_ = 30%, (**d**) R_N_ = 40%, and (**e**) R_N_ = 50%.

**Figure 2:**
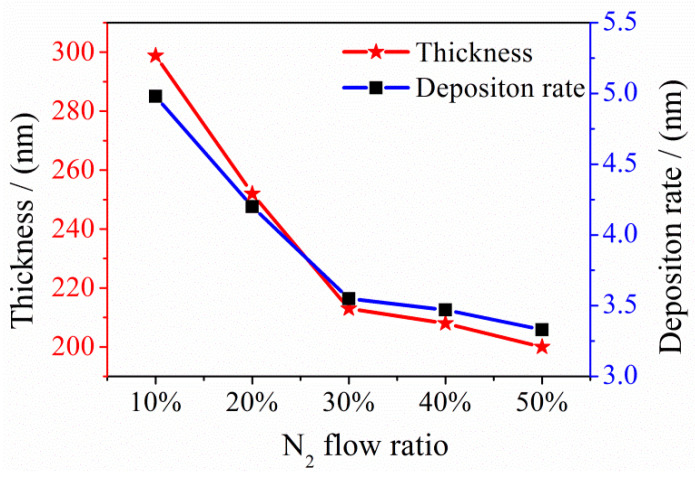
Changes in the thickness and sputtering deposition rate of NbTiAlSiZrN_x_ HEA films.

As the R_N_ increased from 20% to 50% during the preparation process, the thickness decreased from 298.8 nm to 200 nm (Figure 1 and Figure 2).

### 3.2. Assessment of the Film Surface

**Figure 3:**
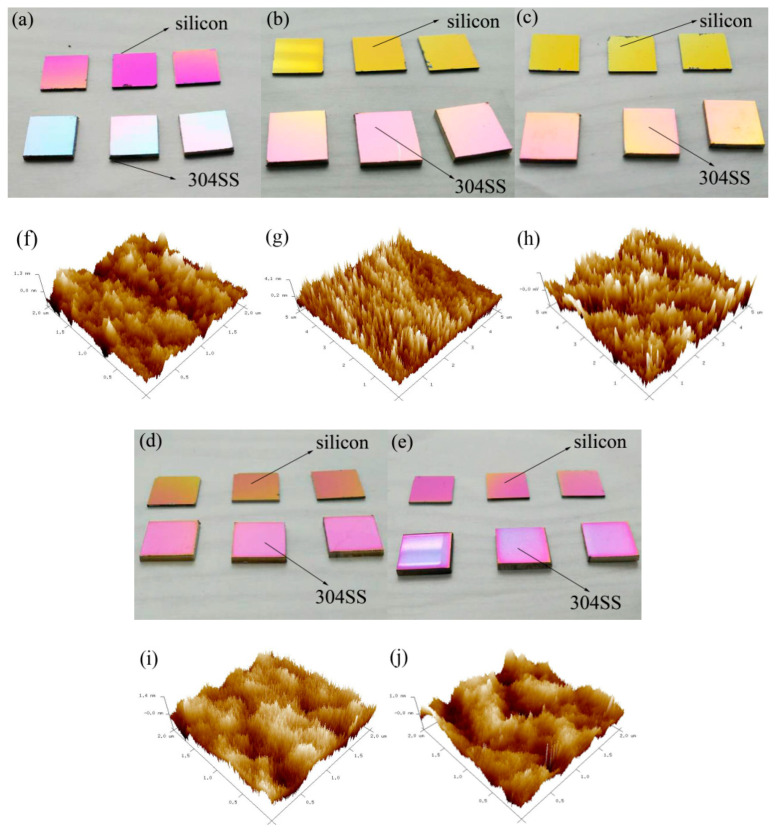
Surface morphology and color of the NbTiAlSiZrN_x_ HEA films as determined by AFM (**a**,**f**) R_N_ = 10%, (**b**,**j**) R_N_ = 20%, (**c**,**h**) R_N_ = 30%, (**d**,**i**) R_N_ = 40%, and (**e**,**g**) R_N_ = 50%.

**Figure 4:**
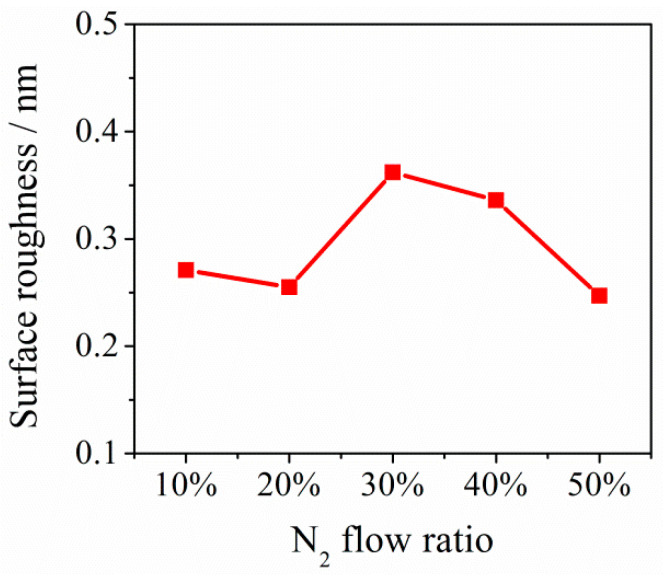
Changes in the roughness values of NbTiAlSiZrN_x_ HEA films.

The surface of NbTiAlSiZrN_x_ HEA films was beautiful, and various base films created different colors. With increasing R_N_, the color of the same base film changed its hue and saturation (Figure 3a–e), whereas film colors had good decorative effects. Surface roughness of NbTiAlSiZrN_x_ HEA films increased with increasing R_N_ from 10 to 50%. However, the non-monotonicity increased from 0.275 nm to 0.365 nm. The roughness was high at R_N_ = 30%. The surface roughness was not uniform across the various regions of the film (Figure 3 and Figure 4).

### 3.3. Assessment of Film Hardness

**Figure 5:**
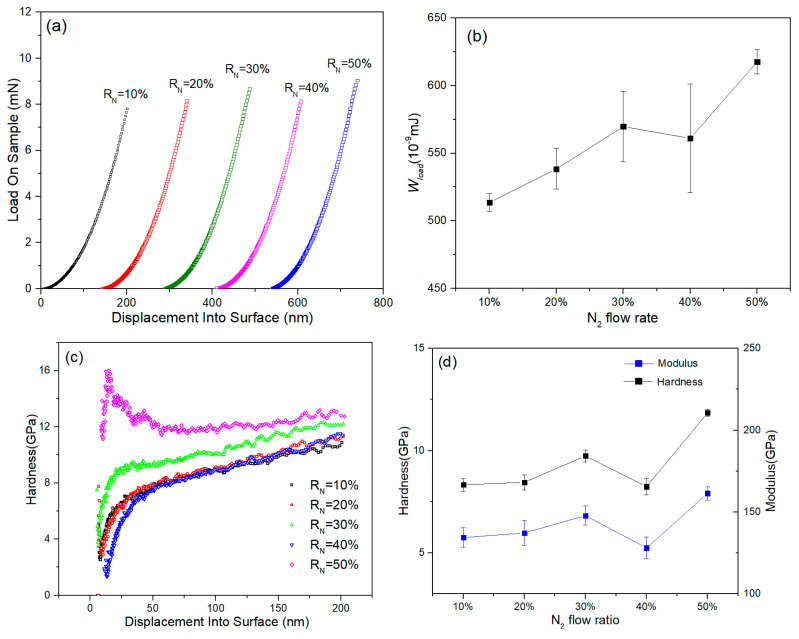
(**a**) Load-Displacement curves of the NbTiAlSiZrN_x_ HEA films; (**b**) changes in the loading energy of the NbTiAlSiZrN_x_ HEA films; (**c**) hardness–displacement curves of NbTiAlSiZrN_x_ HEA films; and (**d**) changes in the hardness and modulus values of NbTiAlSiZrN_x_ HEA films.

The displacement-load curves of various specimens are reported. The increasing trends of the load-displacement curves of the specimens with different R_N_ were similar (Figure 5a). From load-displacement curves, we calculated the loading energy during the lording process using following equation:

(4) W=∫Pdh

where *W_load_* is the loading energy, *h* is the displacement, and *P* is the load on sample. The *W_load_* versus R_N_ curve are exhibited in Figure 5b. *W_load_* represent the energy required for the deformation of HEA films. As is shown in Figure 5b, the *W_load_* increased from 5.13 × 10^−7^ mJ to 5.69 × 10^−7^ mJ when R_N_ ranged from 10–30%, the *W_load_* slightly decreased to 5.61 × 10^−7^ mJ when R_N_ was 40%, then increased to 6.18 × 10^−7^ mJ when R_N_ was 50%. Resistance to external deformation became stronger with the increasing nitrogen content in NbTiAlSiZrN_x_ HEA films. Figure 5c shows the hardness-displacement curves of the specimens with different R_N_, indicating that the hardness gradually increased with the increasing indentation depth in the films. The hardness reached a plateau when the displacement was deeper than 40 nm. To avoid the effect of substrate, we calculated the average hardness and modulus in the depth range of 50–100 nm [37]. The surface hardness and modulus of each sample were high, which increased gradually with increasing R_N_ during preparation, and the hardness had a similar trend (Figure 5d). The lowest hardness of the sample with R_N_ of 10% was 9.7 GPa, whereas the highest modulus of sample with R_N_ of 50% was 184.5 GPa.

### 3.4. Thin Film Corrosion Resistance

**Figure 6:**
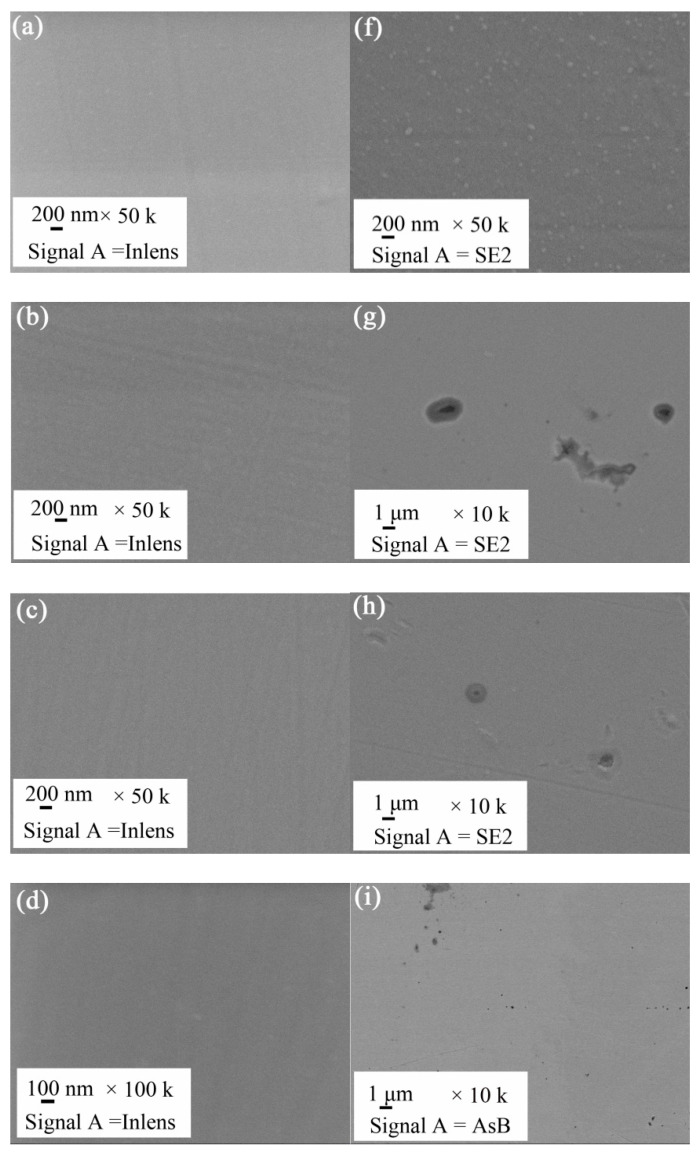

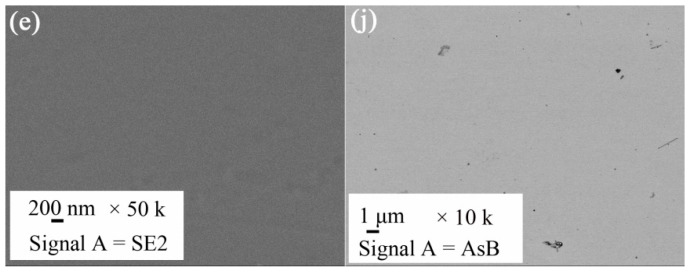
SEM images before and after corrosion of NbTiAlSiZrN_x_ high-entropy films in the 0.5 mol/L H_2_SO_4_ solution. Before: (**a**) R_N_ = 10%, (**b**) R_N_ = 20%, (**c**) R_N_ = 30%, (**d**) R_N_ = 40%, and (**e**) R_N_ = 50%. After: (**f**) R_N_ = 10%, (**g**) R_N_ = 20%, (**h**) R_N_ = 30%, (**i**) R_N_ = 40%, and (**j**) R_N_ = 50%.

**Figure 7:**
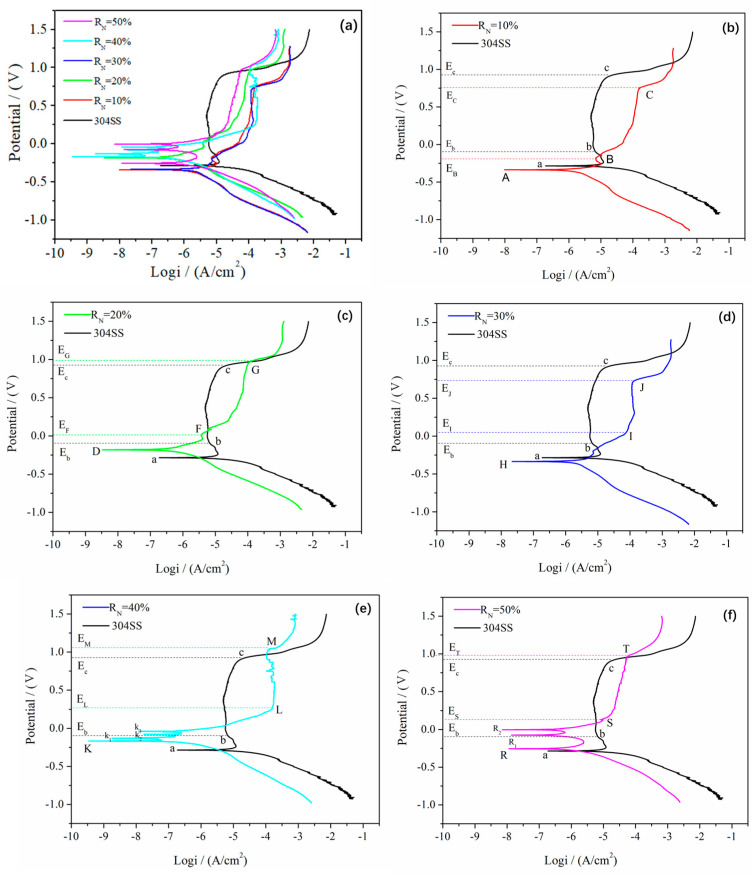
Dynamic potential polarization curves of NbTiAlSiZrN_x_ high-entropy films and 304 stainless steel in 0.5 mol/L H_2_SO_4_ solution. (**a**) all sample, (**b**) R_N_ = 10%, (**c**) R_N_ = 20%, (**d**) R_N_ = 30%, (**e**) R_N_ = 40%, and (**f**) R_N_ = 50%.

**Figure 8:**
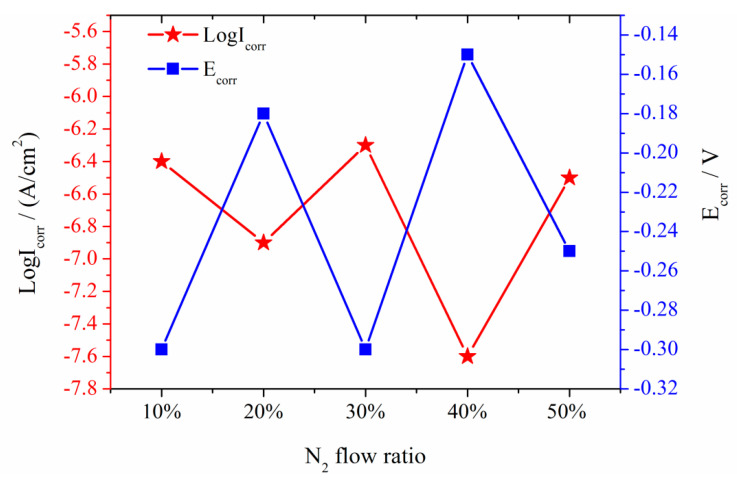
Relationship between the self-corrosion potential and the current of NbTiAlSiZrN_x_ HEA films with the ratio of N_2_ flow.

The corrosion degree of the different thin films, which were prepared with various R_N_ conditions, was different. Corrosion of the sample prepared with 10% R_N_ was slight, and it had almost no pitting corrosion in the 0.5 mol/L H_2_SO_4_ solution (Figure 6f). Non-uniform local pitting corrosion occurred in samples prepared under conditions of R_N_ = 20–50% (Figure 6g–j). However, the most significant non-uniform local pitting corrosion occurred in samples prepared under conditions of R_N_ = 20%. With the increase in R_N_, the number of pits increased, but the maximum size of the corrosion pits decreased (Figure 7). The corrosion potential of 304 SS was approximately −0.25 V, and the corrosion current I_corr_ was approximately 10^−7^ A/cm^2^. The corrosion potential E_corr_ with R_N_ 10% and R_N_ 30% of the samples was approximately −0.3 V, and the corrosion current I_corr_ was approximately 10^−8^ A/cm^2^. The corrosion potential E_corr_ of the samples with 20%, 40%, and 50% R_N_ were higher than those of 304 SS, but the corrosion current I_corr_ was lower than that of 304 SS. The self-corrosion potential and self-corrosion current size at R_N_ = 10% and R_N_ = 30% were similar, and the polarization rate was identical. The self-corrosion potential and corrosion current for the R_N_ = 20% sample were larger, but the polarizability was lower than the R_N_ = 50% sample. The self-corrosion potential of R_N_ = 50% sample was moderate, the self-corrosion current was high, and the polarization rate was low (Figure 8). The passivation interval was not obvious after comparing the NbTiAlSiZrN_x_ HEA thin film with the 304 SS sample, and the passivation interval department start potential and current of NbTiAlSiZrN_x_ HEA thin film were larger than those of the 304 SS sample. The passivation layer breakdown voltage of NbTiAlSiZrN_x_ HEA film samples was lower than that of the 304 SS sample.

### 3.5. Assessment of the Thin Film’s Crystal Structure

**Figure 9:**
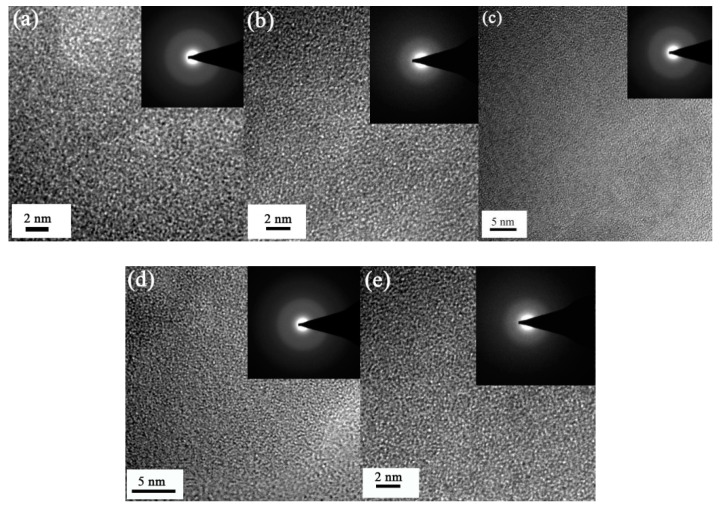
TEM images of NbTiAlSiZrN_x_ thin films at: (**a**) R_N_ = 10%, (**b**) R_N_ = 20%, (**c**) R_N_ = 30%, (**d**) R_N_ = 40%, and (**e**) R_N_ = 50%.

**Figure 10:**
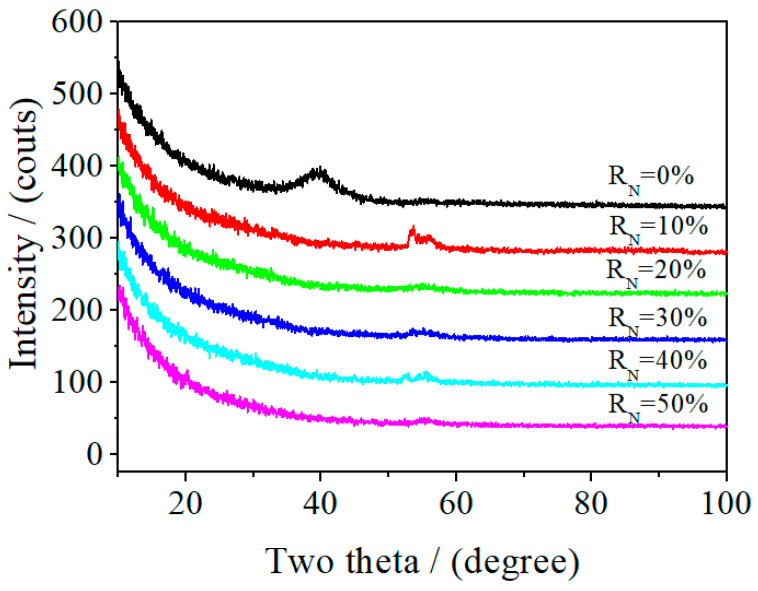
X-ray diffraction (XRD) patterns of NbTiAlSiZrN_x_ thin films.

The microstructure of the polycrystalline phases, such as the grain boundary, were not observed by transmission electron microscopy. The electron diffraction patterns were of diffracting rings, rather than single crystal diffraction spots array of the polycrystalline phases (Figure 9). The X-ray diffraction lines of each sample showed amorphous phase diffraction characteristics. The low and wide protuberant peaks on each line were not the normal diffraction peaks in the NbTiAlSiZrN_x_ HEA films, which may have been caused by inclusions in the films (Figure 10). The results indicate that the different R_N_ samples in the preparation process all had an amorphous structure.

## 4. Discussion

### 4.1. Preparation of NbTiAlSiZrN_x_ HEA Films Using N_2_ Magnetron Sputtering and Thin Film Forming Effect

NbTiAlSiZrN_x_ HEA thin films with an optimal thickness, roughness, and hardness, as well as corrosion resistance, were designed to meet design requirements. The N content in NbTiAlSiZrN_x_ HEA thin films changed with R_N_ during preparation. The test results of NbTiAlSiZr HEA films show that preparing NbTiAlSiZr HEA with proper conditions was feasible. RF magnetron sputtering under the different R_N_ conditions of the process were also feasible to reach the preparation requirements of the NbTiAlSiZrN_x_ HEA thin film.

R_N_ significantly affected the thickness, surface roughness, and color of the prepared thin films in the process. According to the existing theory, increasing the R_N_ value can increase the plasma density near the cathode target and the sputtering yield, so that the sputtered metal atoms are more likely to collide with each other during movement, which reduces the ratio reaching the deposition area on the substrate. By contrast, reducing the kinetic energy of the sputtered atoms colliding with the deposition surface above the substrate and weakening the ratio of strongly adsorbed and bonded to the deposition layer, therefore, need to reduce the thickness of the deposited film during processing. After the plasma density increased, the increased temperature enhanced the thermal radiation on the substrate’s deposition surface. As a result, the thermal kinetic energy of some deposition atoms is increased and then desorbed and volatilized from the deposition surface, thereby reducing the deposition growth rate of the thin film and reducing the accumulation of non-uniform deposition volume during processing. Therefore, the deposited thin film thickness and surface roughness were decreased. 

The sputtered metal atoms and N_2_ crashed and were decomposed by N atoms and N^+^ ions of the ionization under a high degree of vacuum and the high temperature. However, that collision and decomposition on the surface of the initial substrate deposition layer were not uniform. The adsorbed atoms on the surface had been volatized, thereby making the surface roughness and atomic distribution of the sample not uniform. Moreover, the uniformity of color on the surface of the thin film sample was altered, and different sample surfaces were affected by different degrees of color.

### 4.2. Organizational Structure of the NbTiAlSiZrN_x_ HEA Thin Film

Using TEM, we observed no apparent grain boundaries in the thin film samples. The electron diffraction patterns showed amorphous diffraction patterns, and the X-ray diffraction patterns showed no evidence of crystal diffraction, indicating that the prepared NbTiAlSiZrN_x_ HEA thin film was amorphous in structure. N atoms were dispersed in the film and mixed with various metal atoms to form an amorphous phase. 

### 4.3. Performance of the NbTiAlSiZrN_x_ HEA Thin Film

The hardness and modulus of NbTiAlSiZrN_x_ HEA thin film samples increased with the increase of R_N_ during preparation. Hardness of the 50% R_N_ sample reached the highest value at 12.4 GPa, and the modulus reached the highest value at 169 GPa. If the sample had a high hardness, then it had a high wear resistance. The NbTiAlSiZrN_x_ HEA thin film had a beautiful color, which can be used for decorative purposes. However, its roughness affects the decorative effect. The surface roughness of the NbTiAlSiZrN_x_ HEA thin film was small, and it decreased with the decreasing of R_N_. The difference of R_N_ during thin film preparation caused differences in the hardness, surface color, and roughness of the film.

The N_2_ flow rate in the 0.5 mol/L H_2_SO_4_ solution increased during the process. The electrochemical corrosion resistance of the NbTiAlSiZrN_x_ HEA thin film in 0.5 mol/L H_2_SO_4_ solution changed. The corrosion rate of the entropy alloy film was more likely to be eroded compared with 304 SS when HEA thin film with 10% R_N_ and 30% R_N_ compared with 304 SS. However, the corrosion rate of NbTiAlSiZrN_x_ HEA film with R_N_ 20%, 40%, and 50% was lower than that of 304 SS. Furthermore, the passivation stability of the NbTiAlSiZrN_x_ HEA was worse than that of 304 SS. The thin film with a N_2_ flow ratio of 40% had a low polarizability and high corrosion resistance. The film with an N_2_ flow ratio of 30% had a high polarizability and low corrosion resistance. No pitting corrosion occurred in the thin film with a N_2_ flow rate of 10%. The pitting corrosions in the thin films deposited with 20% N_2_ flow rate were different. 

The mixing enthalpy among the elements in NbTiAlSiZr HEA is large and negative, and HEAs possess slow diffusion effect. Therefore, NbTiAlSiZr HEA films easily form amorphous structure under extreme cooling conditions. The amorphous structure will help to improve the corrosion resistance of the HEA film due to the lack of grain boundary corrosion. In addition, the homogeneous distribution of elements in the HEA film also play an important role to improve corrosion resistance [36]. However, the NbTiAlSiZr HEA film is a Cr-free film and it does not contain corrosion-resistant elements, such as Cr, Co, and Ni, with E_corr_ and E_pit_ values lower than those for 304 SS. When a small amount of nitrogen atoms were added into HEAs, some free electrons in HEAs were captured by nitrogen ions. As a result, the free electron concentration decreased, the E_corr_ and E_pit_ of NbTiAlSiZrN_x_ HEA films are close to those of 304 SS (Figure 7b). With the further increase of nitrogen content, E_corr_ and E_pit_ of NbTiAlSiZrN_x_ HEA films with 20% N_2_ flow rate were higher than those of 304 SS (Figure 7c). This is due to the fact that the covalent bond between metal and nitrogen is stronger than the metal bond. The short-range order appeared in NbTiAlSiZrN_x_ HEA films when R_N_ further increased to 30%. E_corr_ and E_pit_ slightly decreased because the migration of nitrogen atoms broke the homogeneous distribution of the NbTiAlSiZrN_x_ HEA film (Figure 7d). Several corrosion potentials appeared on the polarization curve when R_N_ was larger than 40%, the corrosion occurred on several elements of the HEA films (Figure 7d,f).

## 5. Conclusions

The design and preparation of the NbTiAlSiZrN_x_ HEA thin film was feasible using fixed radio frequency pulse magnetron sputtering process parameters and time, and under N_2_ flow rates of 0, 10, 20, 30, 40, and 50% to prepare the NbTiAlSiZrN_x_ HEA thin film on the 304 SS substrate.

When the N_2_ flow rate increased, the sample hardness with 50% R_N_ was 12.4 GPa, the modulus was 169 GPa, the color was beautiful and varied, roughness was small, but the roughness of the thin film prepared under the conditions of 30% N_2_ flow rate was relatively high. The thickness of the thin film gradually reduced from 298.8 nm to 200 nm, and the thin films were amorphous.

N_2_ flow rate increased during the process, the electrochemical corrosion resistance performance of NbTiAlSiZrN_x_ HEA thin film changed in 0.5 mol/L H_2_SO_4_ solution. The N_2_ flow rate of 10% and 30% of the thin film were prone to corrosion more than 304 SS, but its corrosion rate was lower than that of 304 SS. The thin film with N_2_ flow rate of 20, 40, and 50% was more corrosion-resistant than 304 SS. The passivation stability of NbTiAlSiZrN_x_ HEA was worse than that of 304 SS. The thin film with 40% N_2_ flow ratio had a high corrosion resistance and the thin film with 30% N_2_ flow rate had a low corrosion resistance. The corrosion resistance was high, and the film with N_2_ flow rate of 30% had lower corrosion resistance. Moreover, pitting corrosion occurred in the NbTiAlSiZrN_x_ HEA thin film.

## Figures and Tables

**Table 1:** Atomic radii (*r_i_*) of the constituent elements.

Element	Nb	Ti	Al	Si	Zr
*r_i_*	1.48	1.45	1.43	1.34	1.60

**Table 2:** Elemental components of the NbTiAlSiZr high entropy alloy (the atomic percent content).

Element	Nb	Ti	Al	Si	Zr
Nominal content/at. %	20	20	20	20	20
Actual content/at. %	20.56	19.52	21.00	19.38	19.54

Note: the test mode was the EDX area scan.

## References

[1] Braic M., Braic V. (2010). Characteristics of (TiAlCrNbY)C films deposited by reactive magnetron sputtering. Surf. Coat. Technol..

[2] Tsai C., Lai S. (2012). Strong amorphization of high-entropy AlBCrSiTi nitrde film. Thin Solid Films.

[3] Xue W., Zhu Q. (2010). Characterization of ceramic coatings fabricated on zirconium alloy by plasma electrolytic oxidation in silicate electrolyte. Mater. Chem. Phys..

[4] Huang M., Wang Y. (2015). Corrosion Resistance of Fe–Al/Al_2_O_3_ Duplex Coating on Pipeline Steel X80 in Simulated Oil and Gas Well Environment. Surf. Rev. Lett..

[5] Xue K., Niu L. (2007). XPS Analysis of Silicon Oxycarbide Formed on the Surface of Rf-sputter Deposited SiC Thin Films. Key Eng. Mater..

[6] Mondal A.K., Kumar S. (2008). Effect of laser surface treatment on corrosion and wear resistance of ACM720 Mg alloy. Surf. Coat. Technol..

[7] Hsueh H., Shen W. (2012). Effect of nitrogen content and substrate bias on mechanical and corrosion properties of (Al Cr Si Ti Zr)_100-x_N_x_ high-entropy films. Surf. Coat. Technol..

[8] Li Z. (2016). Study on the Design and Preparation of a New Type of Reflective Film with Wide Band Width Angle. Ph.D. Thesis.

[9] Bertolino V., Cavallaro G. (2018). Halloysite nanotubes sandwiched between chitosan layers: Novel bionanocomposites with multilayer structures. New J. Chem..

[10] Cicco D., Asaee Z. (2017). Use of Nanoparticles for Enhancing the Interlaminar Properties of Fiber-Reinforced Composites and Adhesively Bonded Joints-A Review. Nanomaterials.

[11] Duan H., Sun J. Research progress of coating materials and sputtering targets. Proceedings of the 2015 Annual Meeting of the Society of Vacuum Society of Guangdong Province.

[12] Shen W., Tsai M. (2012). Effects of substrate bias on the structure and mechanical properties of (Al_1.5_CrNb_0.5_S_i0.5_Ti)N_x_ coatings. Thin Solid Films.

[13] Zhang Y. (2010). Amorphous and High-Entropy Alloys.

[14] Murty B.S., Yeh J. (2014). High-Entropy Alloys.

[15] Feng X., Tang G. (2013). Chemical state and phase structure of (TaNbTiW)N films prepared by combinatined magnetron sputtering and PBII. Appl. Surf. Sci..

[16] Michael C.G. (2014). Progress in High Entropy Alloys. JOM.

[17] Feng W., Qi Y. (2017). Effects of Short-Range Order on the Magnetic and Mechanical Properties of FeCoNi(AlSi)x High Entropy Alloys. Metals.

[18] Zhai S., Wang W. (2018). Effect of Co and Gd Additions on Microstructures and Properties of FeSiBAlNi High Entropy Alloys. Entropy.

[19] Liu X., Yin H. (2017). Microstructure, Mechanical and Tribological Properties of Oxide Dispersion Strengthened High-Entropy Alloys. Materials.

[20] Klimova M., Stepanov N. (2018). Microstructure and Mechanical Properties Evolution of the Al, C-Containing CoCrFeNiMn-Type High-Entropy Alloy during Cold Rolling. Materials.

[21] Cicala G., Giordano D., Tosto C., Filippone G., Recca A., Blanco I. (2018). Polylactide (PLA) Filaments a Biobased Solution for Additive Manufacturing: Correlating Rheology and Thermomechanical Properties with Printing Quality. Materials.

[22] Senkov O.N., Woodward C. (2014). Microstructure and Propeties of Aluminum-Containing Refractory High-Entropy Alloys. JOM.

[23] Sheng W., Yang X. (2016). Nano-Crystallization of High-Entropy Amorphous NbTiAlSiWxNy Films Prepared by Magnetron Sputtering. Entropy.

[24] Feng X., Zhang J. (2018). Stable nanocrystalline NbMoTaW high-entropy alloy thin films with excellent mechanical and electrical propeies. Mater. Lett..

[25] Zhang S., Wu C. (2014). Synthesis of Laser High Entropy Alloying Coating on The Surface of Single-element Fe Base Alloy. Acta Metall. Sin..

[26] Li P., Chen J. (2015). Research progress of high-entropy alloy coating. Mater. Prot..

[27] Braeckman B.R., Boydens F. (2015). High entropy alloy thin films deposited by magnetron sputtering of powder targets. Thin Solid Films.

[28] Wang F., Liu H. (2014). Advances in ion plating technology. Vacuum.

[29] Huang P., Yeh J. (2009). Effects of nitrogen content on structure and mechanical properties of multi-element. Surf. Coat. Technol..

[30] Chang S., Lin S. (2011). Microstructures and mechanical properties of multi-component (AlCrTiZr)NxCy nanocomposite coatings. Thin Solid Films.

[31] Liu X. (2014). Study on Al_x_FeCrCoNiCu High-Entropy Alloy Coating for Corrosion Protection of Steel Matrix. Ph.D. Thesis.

[32] Sheng W., Yang X. (2018). Amorphous phase stability of NbTiAlSiN_X_ high-entropy films. Rare Metal..

[33] Xing Q., Xia S. (2018). Mechanical properties and thermal stability of (NbTiAlSiZr)Nx high-entropy ceramic films at high temperatures. J. Mater. Res..

[34] Xing Q., Ma J. (2018). High-Throughput Screening Solar-Thermal Conversion Films in a Pseudobinary (Cr, Fe, V)-(Ta, W) System. ACS Comb. Sci..

[35] Zhang Y., Yan X. (2018). Effects of Nitrogen Content on the Structure and Mechanical Properties of (Al0.5CrFeNiTi0.25)Nx High-Entropy Films by Reactive Sputtering. Entropy.

[36] Gao L., Liao W. (2017). Microstructure, Mechanical and Corrosion Behaviors of CoCrFeNiAl_0.3_ High Entropy Alloy (HEA) Films. Coatings.

[37] Feng X., Zhang Y. (2017). Size effects on the mechanical properties of nanocrystalline NbMoTaW refractory high entropy alloy thin films. Int. J. Plast..

